# CXCL12 in Pancreatic Cancer: Its Function and Potential as a Therapeutic Drug Target

**DOI:** 10.3390/cancers14010086

**Published:** 2021-12-24

**Authors:** Shivani Malik, Jill M. Westcott, Rolf A. Brekken, Francis J. Burrows

**Affiliations:** 1 Kura Oncology, Inc., San Diego, CA 92130, USA; smalik@kuraoncology.com; 2 Division of Surgical Oncology, Department of Surgery, and Hamon Center for Therapeutic Oncology Research, University of Texas Southwestern Medical Center, Dallas, TX 75390, USA; jill.westcott@utsouthwestern.edu

**Keywords:** pancreatic cancer, tumor microenvironment, cancer-associated fibroblast, CXCL12, CXCR4

## Abstract

**Simple Summary:**

Pancreatic cancer is a challenging disease to treat effectively. Fibroblasts associated with pancreatic cancer contribute to disease progression by secreting factors that enhance tumor cell survival and help tumor cells avoid detection by the immune system. This overview focuses on a chemokine, CXCL12, produced by cancer-associated fibroblasts and how CXCL12 signaling enhances pancreatic cancer progression by contributing to various hallmarks of cancer including, but not limited to, tumor growth and evasion of immune response. These pro-oncogenic functions of CXCL12 make it an attractive target in pancreatic cancer. We discuss the different approaches in development to therapeutically target CXCL12 and finally propose a novel approach, the use of the farnesyl transferase inhibitor tipifarnib to inhibit CXCL12 expression in pancreatic fibroblasts.

**Abstract:**

Pancreatic ductal adenocarcinoma (PDAC) is a disease with limited therapeutic options and dismal long-term survival. The unique tumor environment of PDAC, consisting of desmoplastic stroma, immune suppressive cells, and activated fibroblasts, contributes to its resistance to therapy. Activated fibroblasts (cancer-associated fibroblasts and pancreatic stellate cells) secrete chemokines and growth factors that support PDAC growth, spread, chemoresistance, and immune evasion. In this review, we focus on one such chemokine, CXCL12, secreted by the cancer-associated fibroblasts and discuss its contribution to several of the classical hallmarks of PDAC and other tumors. We review the various therapeutic approaches in development to target CXCL12 signaling in PDAC. Finally, we propose an unconventional use of tipifarnib, a farnesyl transferase inhibitor, to inhibit CXCL12 production in PDAC.

## 1. Introduction

Pancreatic ductal adenocarcinoma (PDAC), the most common malignancy of the pancreas, accounts for 3% of all cancers and 7% of cancer-related deaths and is expected to claim 48,220 lives in 2021 in the US (American Cancer Society). Despite continued scientific efforts, the 5-year survival rate of all surveillance, epidemiology, and end results (SEER) stages combined remains a dismal 10% (American Cancer Society). Surgery remains the only curative option for PDAC patients. However, over 70% of patients do not qualify for surgical intervention due to locally advanced tumors or their metastatic spread at the time of diagnosis, contributing to the high mortality associated with PDAC [1]. Chemotherapy in the forms of FOLFIRINOX (a cocktail of 5-fluorouracil, irinotecan, and oxaliplatin) and a combination of gemcitabine with nab-paclitaxel (nanoparticle albumin-bound paclitaxel) remain the mainstay of metastatic PDAC treatment [2]. Even with these therapies, the 5-year survival rates for patients with regionally advanced and metastatic disease stand at 12% and 3%, respectively.

A major obstacle to effectively treating PDAC is attributable to its unique tumor microenvironment (TME). Unlike many other solid cancers, an overwhelmingly large proportion—sometimes as much as 80%—of the total tumor volume in PDAC is comprised of nontumor stroma [3]. This dense, fibrous stroma, referred to as desmoplasia, contains extracellular matrix (ECM) proteins as well as stromal cells (including immune cells such as regulatory T cells (Tregs), myeloid-derived suppressor cells (MDSCs), and tumor-associated macrophages (TAMs)), fibroblasts (such as pancreatic stellate cells (PSCs) and cancer-associated fibroblasts (CAFs)), and endothelial cells. The ECM is composed of collagens, laminin, fibronectin, glycosaminoglycans, and other soluble factors [4]. Together, the cellular and structural components of the TME function as a dynamic network that drives tumor cell growth, invasion, metastasis, and therapeutic resistance.

In normal pancreatic tissue, the structural components of the ECM function as a scaffold and signaling matrix to maintain tissue homeostasis, while resident fibroblasts and quiescent PSCs conserve connective tissue organization and immune cells engage in immune surveillance. However, upon tumor initiation, cancer cells manipulate the surrounding microenvironment to their own benefit by shifting these components into a state that allow tumorigenesis. Cancer cells can alter the TME directly (via the secretion of signaling molecules) as well as indirectly (through the resulting hypoxia and oxidative stress), the effects of which include fibroblast/PSC activation and recruitment, blood vessel formation, and the initiation of an inflammatory response [5]. Upon their activation, PSCs show increased proliferation and migration and assume a myofibroblast-like phenotype that involves the expression of alpha-smooth muscle actin (α-SMA), fibroblast-specific protein-1 (FSP-1), and fibroblast activation protein-alpha (FAP-α) [6]. Activated PSCs (aPSCs) also show excessive deposition of ECM proteins, resulting in increased interstitial pressure and tissue rigidity, which can contribute to impaired drug delivery and increased cancer cell migration [7,8]. Furthermore, aPSCs secrete chemokines, growth factors, and other soluble proteins that can enhance tumor cell growth and migration, promote angiogenesis, and induce immune evasion.

**Figure 1:**
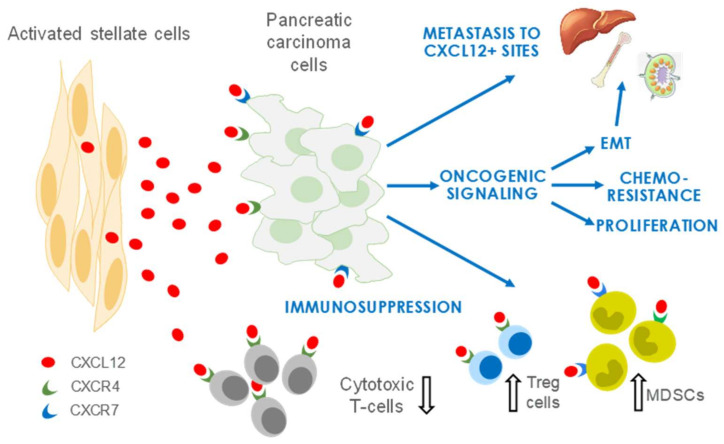
Roles of stroma/stellate cells in PDAC. CXCL12 secreted predominantly by activated stellate cells/CAFs promotes an environment conducive to PDAC growth and metastasis. CXCL12 also has crucial functions in immune evasion and development of resistance to chemotherapies. EMT, epithelial-to-mesenchymal transition; Treg, regulatory T cell; MDSCs, myeloid-derived suppressor cells.

CXCL12, a member of the CXC family of chemokines, is secreted by the activated fibroblasts (for which we use the terms aPSCs and CAFs interchangeably in this review) of the TME and is a crucial mediator reported to contribute to growth and metastasis in PDAC and several other solid tumors, including head and neck squamous cell carcinoma (HNSCC) and breast, ovarian, and colorectal carcinomas [9,10,11]. CXCL12 has a pervasive influence in PDAC by increasing proliferation, enhancing invasion and metastasis, and promoting chemoresistance and immune evasion of tumor cells (Figure 1). The two receptors of CXCL12, CXC receptor 4 (CXCR4) and atypical chemokine receptor 3 (AKRC3, also known as CXCR7), are expressed on PDAC cells, and an elevated expression of CXCR4 is associated with a poor prognosis in several cancer types [12]. In this hypothesis paper, we discuss the oncogenic functions of CXCL12 and its potential as a therapeutic target in PDAC.

## 2. Materials and Methods

### 2.1. RNA Extraction and qPCR

For RNA studies, 20,000 human pancreatic primary stellate cells were plated on a 96-well plate on day one. Stellate cells were obtained from ScienCell (catalog #3830, Carlsbad, CA, USA) and grown according to vendor instructions. Cells were treated with tipifarnib on day two and collected for RNA extraction and qPCR on day five using a Cells-to-Ct 1-step TaqMan kit (A25603, Thermo Fisher Scientific, Carlsbad, CA, USA). TaqMan primer-probes were purchased from Thermo Fisher Scientific as follows: *CXCL12*- HS00171022_m1; *GAPDH*-HS02786624_g1. Data were analyzed using the Delta-Delta Ct method in Microsoft Excel. GAPDH was used as the housekeeping control gene to normalize *CXCL12* gene expression. Statistical significance was calculated using two-tailed *t*-test function in Microsoft Excel.

### 2.2. ELISA

For ELISA protein studies, 150,000 human pancreatic primary stellate cells were plated on six-well plates on day one. Cells were treated with different doses of tipifarnib (in fresh media) using serial dilution method on day two. Supernatants for ELISA were collected on day five. ELISA was performed using Abcam kit 100637 following manufacturer’s protocols. Dose–response curves were plotted using GraphPad Prism 9.

### 2.3. TCGA Data Analysis

CXCL12 expression data and KRAS mutation data were downloaded from cbioportal using the pancreatic adenocarcinoma (TCGA, Firehose Legacy) dataset. A correlation scatter plot was made on Microsoft Excel. Pearson correlation coefficient (r) and two-tailed statistical significance at 99% were calculated using GraphPad Prism assuming that the data were sampled from a Gaussian distribution.

### 2.4. PDX Mouse Studies

Mouse studies were performed at Crown Bio (Crown Bioscience SPF facility, Global Headquarters, San Diego, CA, USA). All protocol and any amendment(s) involving the care and use of animals were approved by the Institutional Animal IACUC of CrownBio prior to conducting the study, as previously described [13]. Fresh tumor tissues from mice bearing established primary human cancer tissues were harvested and cut into small pieces (approximately 2–3 mm in diameter). These tumor fragments were implanted subcutaneously into BALB/c nu/nu mice. The inoculated grafts were allowed to establish to 250–350 mm^3^, following which the animals were randomized into groups of two and treated orally BID with vehicle or tipifarnib (60 mg/kg) for 25–35 days. Tumor size was measured by caliper twice weekly. Tumor volumes were calculated in mm^3^ using the formula Volume = (Tumor Length × Tumor Width × Tumor Width)/2, where tumor length is the longest tumor dimension and tumor width is the longest tumor dimension perpendicular to length.

## 3. CXCL12 Signaling

Chemokines (from “kinos”, the Greek word for movement) are low-molecular-weight secreted proteins belonging to the family of small cytokines. Chemokines are classified into four groups—C, CC, CXC, and CX3C—based on the position of the conserved cysteine residues that are crucial for their three-dimensional folding [14]. Chemokines are key mediators of concentration-dependent cellular migration, a process called chemotaxis. They signal through G protein-coupled seven-span transmembrane receptors to exert control over diverse biological processes, such as growth, survival, migration, adhesion, and cytoskeletal reorganization. CXCL12, a homeostatic chemokine, was first described as a protein secreted by bone marrow stromal cells (hence the alternative name for CXCL12, stromal cell-derived factor-1 (SDF-1)), that function as a strong chemoattractant for lymphocytes and monocytes/macrophages [15]. Almost concurrently, CXCL12 was found to be essential for B-cell lymphopoiesis and bone marrow myelopoiesis during embryogenesis (hence, another alternative name for CXCL12, pre–B-cell growth factor) [16]. Its cognate receptor, CXCR4, is expressed on most leukocyte subsets and lymphoid cells of the bone marrow, thymus, and lymph nodes [17]. In addition to CXCL12, other factors such as macrophage migration inhibitory factor (MIF), pancreatic adenocarcinoma upregulated factor (PAUF), and ubiquitin have been reported to activate the CXCR4 receptor [18,19,20]. However, there is little known about non-CXCL12 ligand-mediated signaling through CXCR4, and most of our current understanding of signaling through CXCR4 is derived from its activation by CXCL12.

Upon binding to CXCR4, CXCL12 activates signaling cascades emanating from activated G proteins [21,22,23,24]. In the absence of CXCL12, CXCR4 is coupled to GDP-bound G_i_α, which forms an inactive trimeric G protein with Gβγ. Upon CXCL12 stimulation, CXCR4 undergoes a conformational shift that favors the exchange of GDP for GTP, releasing GTP-bound G_i_α from Gβγ. Free GTP-G_i_α and Gβγ then activate different downstream signaling pathways. GTP-G_i_α inhibits adenylyl cyclase and activates mitogen-activated protein kinase (MAPK) signaling, promoting cell proliferation and migration. Gβγ induces phospholipase C (PLC)/protein kinase C (PKC)-Ca^2+^ signaling to enhance chemotaxis and activates the phosphatidylinositol-3-kinase (PI3K) pathway to enhance cell survival [21,22,23,24]. While the majority of the stimulated CXCR4 signaling is G protein-dependent, CXCR4 can also signal in a G protein-independent fashion. For example, CXCL12 stimulation can activate the JAK/STAT pathway, in part independently of G-protein signaling [25]. Additionally, arrestin-2 and -3 also enhance CXCR4-activated MAPK signaling in a G protein-independent manner, and arrestin-3 has been reported to activate p38 MAP kinase directly to promote cell migration [26,27]. Some researchers have suggested that the homodimerization of CXCR4 receptors is required for G protein-independent alternative signaling pathways [25].

While the signaling pathways emanating from CXCR4 are relatively well-characterized, the contribution of CXCR7 is just beginning to be appreciated. Unlike typical chemokine receptors, CXCR7 is not coupled to G proteins. Instead, CXCR7 functions primarily to refine or modify CXCL12-induced signaling through CXCR4 by scavenging extracellular CXCL12 and thus limiting signaling through CXCR4 [28,29]. CXCR7 can also heterodimerize with CXCR4 to regulate CXCL12 signaling [30,31]. It is not entirely clear how the heterodimer modulates CXCL12 signaling, with reports demonstrating that the heterodimer may function as an enhancer or an inhibitor of CXCR4-driven G-protein activation [30,31]. It was later shown that the CXCR4-CXCR7 heterodimer recruits β-arrestin, resulting in a preferential activation of β-arrestin-associated pathways compared to canonical G-protein signaling [32]. In addition to signaling through β-arrestin as a heterodimer, CXCR7 can independently signal through this pathway to activate MAPK and induce cellular migration [33,34,35]. The interplay between CXCR4 and CXCR7 may be important in PDAC since CXCR4 and CXCR7 are often co-expressed in human pancreatic cancer cell lines and tissues [36,37].

In the sections below, we describe how the canonical functions of CXCL12 are misappropriated in PDAC to mediate pro-oncogenic activities, including cancer cell survival and spread, immune suppression, and chemoresistance.

## 4. CXCL12 Signaling in PDAC

As described above, PDAC is a stroma-rich cancer. The activated fibroblasts (aPSCs and/or CAFs) of the TME are the predominant source of CXCL12 that facilitates the acquisition, maintenance, and enhancement of several cancer hallmark traits [2,38]. Studies spanning over the last decade and a half using in vitro co-culture and in vivo models along with employment of the CXCR4 inhibitor AMD3100 (plerixafor; trade name, Mozobil) has enabled a deeper understanding of the signaling pathways involved in the acquisition of these traits as discussed below.

### 4.1. CXCL12 Promotes PDAC Survival and Proliferation Signaling

CXCL12 promotes cell survival and expansion via the stimulation of dominant oncogenic RAS-MAPK and PI3K-AKT signaling pathways. Given that aPSCs form the bulk of the PDAC stroma, most studies have evaluated the effect of PSCs on PDAC cells to understand the PDAC stromal–tumor interactions. Early understanding of interactions between PSCs and PDAC cells comes from two-dimensional tumor cell line studies that showed the effect of PSC-conditioned media on tumor cell growth and survival. In one such study [39], Marchesi et al. noted that PSCs increased the proliferation, invasion, and transendothelial migration of CXCR4^+^ pancreatic cancer cell lines and protected the tumor cells from apoptosis in vitro. Further, PSCs also enhanced the growth rate of several PDAC models in vivo in the subcutaneous site [40]. Importantly, Hwang et al. [41] further substantiated these findings by demonstrating that PSCs reduced latency periods and enhanced tumor growth and metastasis in an orthotopic model of PDAC. The co-implantation of a human pancreatic tumor cell line with human PSCs in mouse pancreas resulted in increased take rates and higher tumor burdens compared with tumor cells alone. PSCs also enhanced the rate of metastasis to the lymphatic, hepatic, and peritoneal sites [41]. Importantly, several other reports have corroborated the supportive contribution of CXCL12 to PDAC growth and migration in vitro [42,43,44], reinforcing the concept that CXCL12 production by PSCs might underlie their facilitation of PDAC growth and progression. Biochemical analyses further demonstrate that CXCL12 supports KRAS-induced MAPK and AKT signaling to promote tumor cell survival and proliferation [36,43,45,46,47]. Interestingly, an ERK inhibitor suppressed cell–cell interactions in an in vitro co-culture model of PDAC with PSCs, suggesting that ERK signaling is important in tumor and stellate cells [48]. Most of these studies implicate CXCR4 as the primary mediator in CXCL12-induced activation of the MAPK and AKT pathways. While the function of CXCR7 is less well-characterized, CXCR7 has been shown to contribute to increased MAPK signaling in receptor positive PDAC cell lines [36]. Similar to PDAC, CXCL12 also amplifies MAPK signaling in breast, colon, head and neck, and esophageal cancers [49,50,51,52].

### 4.2. CXCL12 Signaling Promotes Immune Evasion

PDAC so far has remained disappointingly refractory to immunotherapy targeting immunological checkpoints such as cytotoxic T-lymphocyte-associated antigen 4 (CTLA-4) and programmed cell death protein-1 and its ligand (PD-1/PD-L1). Those same immuno-oncology drugs are now approved for several other solid tumors, including cancers of the head and neck, bladder, and kidney [53]. Thus, there is great interest in understanding the biological mechanisms underlying PDAC resistance to immunotherapy [54].

The dense stroma of PDAC has a complex, pleotropic immunomodulatory function in PDAC progression, and understanding the stromal-immune cell interaction is crucial in designing immune therapies for this disease. Most of the understanding of the PDAC immune microenvironment is based on findings from genetically engineered mouse models (GEMMs) that indicate that PDAC is a “cold” or “non-inflamed” tumor characterized by the absence of antigen-specific T-cell responses and elevated levels of immunosuppressive MDSCs and Tregs [55,56,57]. While such a T-cell exclusion has been described in human PDAC, multiple studies reported that a fraction (16–35%) of patients exhibited CD4^+^ and CD8^+^ T-cell infiltration and that higher numbers of T cells in juxtatumoral stroma correlated with better survival [58,59,60,61,62,63]. The “immune ignorance” model views PDAC as a cold tumor, thus favoring priming the initial T-cell response as an immunotherapy strategy [64]. Conversely, the “immune suppressive” model of PDAC advocates for the utility of enhancing T-cell activation with checkpoint inhibitors such as monoclonal antibodies to CTLA-4 or PD-1/PD-L1. Evidence supporting each model exists, and the best strategy perhaps depends on the immune landscape of the individual tumor. Regardless, activated stromal fibroblasts are crucial immune modulatory cells. For example, the depletion of CXCL12-producing fibroblast activation protein-α (FAP^+^) CAFs allowed for immunological control of tumor growth in *KPC* (*Pdx1^Cre^, Kras^LSL-G12D^,* and *Trp53^LSL-R172H^*) mice and a combination of CAF depletion or CXCR4 inhibition with AMD3100 with anti–PD-L1 resulted in tumor regressions [65,66]. This synergistic response was recapitulated in viable resected human PDAC slices by Seo and coworkers, who elegantly demonstrated improved homing of CD8^+^ T cells to juxtatumoral stromal regions and enhanced CD8^+^ T cell-mediated antitumor activity by combined blockade of CXCR4 and PD-1 [58]. Furthermore, a recent study showed that PDAC excludes T cells and resists inhibitors of PD-1 checkpoints when cancer cells are coated with covalent heterodimers of CXCL12 and keratin 19 (KRT19) formed by transglutaminase-2 (TGM2). Interrupting the expression of KRT19 or TGM2 in mouse PDAC allowed the infiltration of T cells and sensitized the response to anti–PD-1 agents [67].

### 4.3. CXCL12 Signaling Promotes Angiogenesis

Angiogenesis is the process of new blood vessel development from pre-existing vessels. Tumors hijack this cellular process for their sustenance. Fibroblast-derived CXCL12 in concert with pancreatic tumor-derived CXCL8 has been shown to promote proliferation, invasion, and tube formation of endothelial cells in vitro [68]. Furthermore, in a subcutaneous mouse model of PDAC, CXCR4 blockade by AMD3100 reduced intratumor blood flow and tumor vascular density, supporting that CXCL12 can promote angiogenesis [69]. Together, these studies suggest that CXCL12 supports angiogenesis in PDAC.

### 4.4. CXCL12 Signaling Promotes Chemoresistance

The nucleoside analog gemcitabine remains a cornerstone of PDAC therapy, but inherent or acquired resistance to gemcitabine limits its benefit to patients. As a result, considerable effort has been expended to decipher the underlying mechanisms of resistance to gemcitabine. Stroma-tumor interactions in general and CXCL12-CXCR4 signaling in particular contribute significantly to drug resistance in PDAC. The CXCL12/CXCR4 axis promotes innate gemcitabine resistance by activating pro-survival pathways and contributes to acquired resistance via the upregulation of CXCR4 expression. CXCL12 protects PDAC cells from the cytotoxic effects of gemcitabine in part by NF-kB-dependent anti-apoptotic signaling, promoting the expression of survival proteins such as Bcl-2, Bcl-xL, and survivin [70]. CXCL12 also induces an autocrine IL-6 secretion loop in PDAC cells to further enhance chemoresistance [71]. Gemcitabine also counterproductively increases CXCR4 and CXCR7 expression in PDAC cells, which in turn enhances CXCL12 production by stromal cells and renders PDAC cells more invasive and resistant to gemcitabine [72,73]. Consistent with a chemoprotective role of CXCL12 signaling, its disruption by AMD3100 has sensitized PDAC to gemcitabine in vitro and in vivo [74]. AMD3100 is also known to sensitize prostate cancer cells to docetaxel and colon cancer cells to 5-fluorouracil [75,76,77], implying that the blockade of CXCL12 signaling could have wider application as a chemosensitization strategy.

## 5. Therapeutic Targeting of CXCL12/CXCR4 in Pancreatic Cancer

The numerous pro-tumorigenic functions of CXCL12/CXCR4 signaling described above render this pathway as a potentially valuable therapeutic target in PDAC. CAFs are the predominant source of CXCL12 in PDAC. The most well-explored strategy to block CXCL12 signaling is through inhibition of its receptor CXCR4 with AMD3100, approved by the FDA in 2008 as a mobilizer of CD34^+^ hematopoietic cells from the bone marrow into circulation. In addition to non-peptide small molecule inhibitors such as AMD3100, several other classes of drugs have been developed to inhibit CXCR4, including (a) small modified peptide CXCR4 antagonists (T140 and its analogs, TN 14003/BKT140); (b) antibodies to CXCR4 (BMS-936564/MDX-1338); and (c) microRNAs such as miR-302a, miR-9, miR-204-5p, and miR-126 [24].

A distinct emerging strategy to target the CXCL12/CXCR4 pathway is to target CXCL12 itself. Noxxon Pharma is developing NOX-A12, a pegylated L-oligoribonucleotide that binds and neutralizes CXCL12 and has shown some early promise in the clinic, but most CXCL12-directed therapies are far from clinical application [78,79].

**Figure 2:**
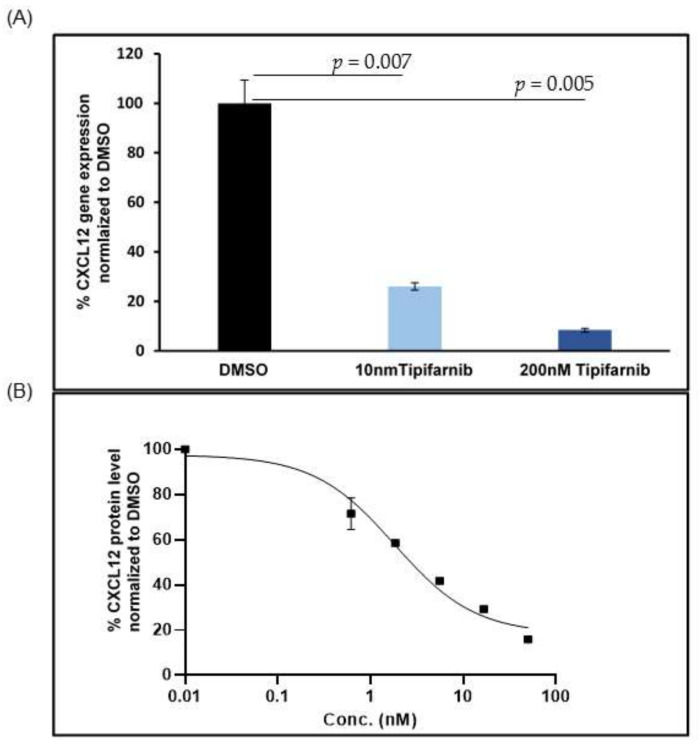
Tipifarnib inhibits CXCL12 expression in human pancreatic stellate cells. (**A**) Response of CXCL12 RNA level following 3 days of tipifarnib treatment as measured by quantitative PCR; (**B**) response of CXCL12 protein level to 3 days of tipifarnib treatment as measured by ELISA. Two-tailed *t*-test *p* values are depicted on the plots in (**A**).

Here, we propose that CXCL12 signaling in solid tumors can be effectively silenced with a well-tolerated drug with a large safety database that is currently in late-stage development for cancer. Tipifarnib, a highly selective and potent farnesyltransferase inhibitor, originally developed by Janssen as a KRAS inhibitor in the early 2000s, has displayed sporadic clinical activity in several unstratified cancer patient populations. The geranylgeranylation pathway rescues KRAS and NRAS when farnesylation is blocked [80], excluding the only pre-identified large target patient populations. The paucity of sophisticated screening technologies at the time that the studies were conducted prevented the drug from being matched with other, more appropriate biomarkers, leading to its eventual failure in the clinic. The advent of advanced sequencing methodology now enables high-throughput screening of patients for relatively rare mutations, prompting an effort to take the drug back into development, this time as a targeted therapeutic against HRAS, the only RAS isoform critically dependent on farnesylation for its activity. Early clinical data [81] were very promising, leading Kura Oncology to initiate a pivotal phase 2 trial in relapsed/refractory HRAS-mutant HNSCC in 2018. Unexpectedly, we found that tipifarnib potently inhibited CXCL12 gene expression in human PSCs (Figure 2A), suggesting that the drug can silence CXCL12 signaling in vivo. In the sections below, we focus on approaches to targeting the CXCL12 axis in pancreatic cancer.

### 5.1. CXCR4 Antagonists in Pancreatic Cancer

The CXCR4 inhibitor AMD3100 was originally developed for treating acquired immune deficiency syndrome (AIDS). The T-cell tropic HIV-1 strain uses CXCR4 as a co-entry receptor to infect T cells. A CXCR4 inhibitor would inhibit this viral envelope-CXCR4 interaction and aid in limiting HIV-1 infection [82]. The serendipitous observation that it increased white blood cell counts in healthy volunteers in a phase 1 trial led to its subsequent development as a mobilizer of progenitor and stem cells from the bone marrow. The physiological role of CXCL12 in bone marrow homing explains this clinical observation. CXCL12 is crucial in bone marrow retention of CXCR4^+^ hematopoietic stem cells (HSCs), where it functions as a chemoattractant, and the blockade of CXCR4 thus leads to an egress of HSCs into the peripheral blood. In 2008, AMD3100 (trade name, Mozobil) was approved as a stimulant for the collection of HSCs and their subsequent autologous transplantation in patients with non-Hodgkin’s lymphoma and multiple myeloma, with the drug being used in combination with a granulocyte colony stimulating factor (Neupogen). The ability of Mozobil to mobilize HSCs out of bone marrow also made it an attractive therapy to test in leukemia. Leukemia stem cells escape the cell cycle-dependent cytotoxicity of chemotherapy because they are maintained in a quiescent state in protective bone marrow niches, suggesting that Mozobil could potentially chemosensitize circulating blasts by blocking bone marrow homing, and encouraging preliminary results were observed for acute myeloid leukemia [83]. These findings prompted the exploration of Mozobil use in solid cancers where CXCL12 is implicated prominently in their biology, including PDAC.

CXCR4 is frequently expressed at high levels in pancreatic tumor cell lines, particularly those derived from metastatic lesions. CXCL12 promotes proliferation, chemotaxis, and invasion of these CXCR4^+^ PDAC cells, and these oncogenic effects can be blocked by AMD3100 or neutralizing antibodies to CXCR4 or CXCL12 [39,84]. Although the predominant source of CXCL12 in PDAC is aPSCs, PDAC cells can also produce the chemokine. Interestingly, AMD3100 reduced the proliferation of Hs766t, a CXCL12^+^ KRAS wild-type (WT) pancreatic tumor cell line, suggesting an autocrine regulatory loop that may be at play in some pancreatic tumors [39]. In addition to potentiating growth and migration, CXCL12 has been shown to protect pancreatic cancer cells from the cytotoxic effects of the PDAC standard-of-care drug gemcitabine [70,74].

As discussed above, the stroma-rich PDAC microenvironment is crucial in mediating immune suppression by modulating local immune responses. Given the observation that AMD3100 in combination with an anti-PD-L1 antibody can lead to effective clearance of mouse PDAC [65], Jodrell et al. tested AMD3100 in a phase 1 trial (NCT02179970). AMD3100 was administered intravenously continuously for 1 week, and biopsies were collected pre- and post-AMD3100 infusion. Transcriptional analyses of these paired biopsies of metastases from microsatellite stable colorectal cancer and pancreatic cancer showed that CXCR4 inhibition induced an integrated immune response involving multiple mediators of innate and adaptive immune responses [85,86]. In addition, a recent study using pancreatic patient samples revealed a mobilization of CD8^+^ T cells in juxtatumoral areas following CXCR4 inhibition and increased tumor cell killing when combined with PD-L1 inhibition [58]. Though preliminary, these findings suggest that the combination of CXCR4 inhibition with immune modulatory and cytotoxic drugs may be an attractive therapeutic strategy in PDAC. Pertinently, there are active trials testing this strategy in metastatic pancreatic cancer patients using BL-8040 (motixafortide), a short synthetic peptide antagonist of CXCR4, in combination with pembrolizumab (NCT02907099) or chemotherapy (NCT02826486 and NCT03193190).

### 5.2. CXCL12 Antagonists in Pancreatic Cancer

As described above, early strategies for targeting CXCL12 signaling centered upon CXCR4 antagonists, but recently the targeting of CXCL12 itself has gained some traction as an alternative approach. In pancreatic cancer, where CXCR4 and CXCR7 receptors are commonly co-expressed on tumor cells, blocking or depleting CXCL12 may be a more effective strategy.

NOX-A12 is a novel RNA aptamer that binds CXCL12 in two key positions, blocking binding of the chemokine to its receptors and dislodging bound CXCL12 from cell surfaces [79]. NOX-A12 has synergized with PD-1 blockade by enhancing T-cell infiltration in preclinical models [87], leading to an exploratory phase 1B study with a small cohort of 11 colorectal and 9 pancreatic cancer patients, where a combination of NOX-A12 with the PD-1 inhibitor pembrolizumab induced T helper type 1 (Th1) immune responses and prolonged disease stabilization in a minority of patients, supporting previous findings that the CXCL12/CXCR4 axis is important in immune evasion in pancreatic cancer [88].

We recently found that tipifarnib, a farnesyltransferase inhibitor, inhibits CXCL12 gene expression in activated fibroblasts. Tipifarnib effectively reduces CXCL12 protein levels in activated human PSCs at a concentration of 10 nM (Figure 2B). This offers an opportunity to repurpose tipifarnib to target CXCL12 in PDAC. This unanticipated finding and the emerging role of CXCL12 in PDAC also prompted us to reevaluate tipifarnib clinical activity in PDAC in the context of CXCL12-related biomarkers. The INT-11 study (NCT00005648) was a placebo-controlled phase 3 trial evaluating the efficacy of gemcitabine compared with gemcitabine plus tipifarnib in an unselected cohort of PDAC patients that failed to demonstrate the clinical activity of tipifarnib, and the approach was abandoned. However, retrospective subset analysis provides several interesting associations. A common feature of PDAC at presentation is abdominal pain due to perineural tumor invasion. CXCL12 secreted by PDAC lesions attracts Schwann cells (SCs) of nearby nerves to migrate into tumor deposits. This neuronal migration paradoxically attenuates PDAC-associated pain by the downregulation of pain-associated genes in SCs [89]. A corollary to this observation is that PDAC patients with high levels of CXCL12 attract more SCs and hence experience less pain compared with patients with low levels of CXCL12. Considering the association between high CXCL12 expression and the attenuation of pain, we hypothesized that the absence of reported abdominal pain (equating to high CXCL12 levels) could be a surrogate marker for clinical benefit from tipifarnib. We tested this hypothesis with a retrospective analysis of the INT-11 trial comparing the survival of patients who received gemcitabine + placebo (GP) with gemcitabine + tipifarnib (GT) based on the presence or absence of reported pain. An absence of abdominal pain at study entry was associated with higher median survival only in the GT group (pain vs. no pain: 5.9 months vs. 10.2 months; HR = 0.52; *p* < 0.0001) with no effect in the GP group (pain vs. no pain: 6.0 months vs. 6.1 months). This analysis supports the idea that tipifarnib-based regimens may benefit PDAC patients with high CXCL12 levels by virtue of its CXCL12 inhibitory property [90].

## 6. Discussion

PDAC is a deadly disease with few treatment options. The prominent CAF component of PDAC is a rich source of factors that maintain a permissive environment for tumor growth and spread, and enhances resistance to therapeutic drugs of several classes. The CAF product CXCL12 is an oncogenic chemokine that promotes many of the classical hallmarks of cancer and, as such, the cumulative effect of inhibiting it could provide significant clinical benefit.

Evidence from early clinical studies combining CXCL12 signaling inhibitors with immunotherapies have provided proof-of-concept for their therapeutic activation of immunity in PDAC. Small cohort trials evaluating anti–PD-L1 antibodies combined with AMD3100, BL-8040, or NOX-A12 have demonstrated that the inhibition of CXCL12 signaling can potentiate immune responses in PDAC. While the exact underlying mechanisms are unclear, CXCL12 inhibition may serve as an immune stimulant, immune potentiator, and/or microenvironment modifier. Preclinical studies from two independent groups have demonstrated that co-administration of AMD3100 with PD-1/PD-L1 antibodies markedly enhanced T-cell infiltration into tumor sites [58,65]. How does CXCL12 exclude T cells from PDAC sites in the first place? T-cell exclusion could be mediated by fugetaxis—an active movement of T cells away from a high concentration of CXCL12 coating the tumor cells [91]—or T-cell apoptosis similar to that observed by the engagement of CXCR4 with the gp120 coat protein of HIV [92]. Alternatively, the heterogeneity and spatial arrangement of CAFs in relation to tumor cell nests may also be an important contributing factor in establishing an immunosuppressive TME.

For example, elevated CXCL12 levels in stroma-rich areas of the tumor mass that lack tumor cell nests could generate a concentration gradient attracting T cells away from the malignant cells themselves. Indeed, CAFs appear to limit the access of CD8^+^ cells to juxtatumoral stromal areas in PDAC [93]. Intriguingly, a recent clinical study of NOX-A12 revealed that drug treatment significantly reduced the average distance between T cells and tumor cells in PDAC biopsies [88]. The stroma can thus function as a physical and a dynamic chemical barrier working actively to keep antitumor immune cell types (e.g., CD8^+^ T cells) out while permitting the migration of immunosuppressive cells such as MDSCs and mast cells. CXCL12 inhibition in these tumors may thus provide benefits by reducing the contribution of the immunosuppressive cells and by enhancing T-cell infiltration. Priming T-cell immune responses by activating CD40 or dendritic cell activation may also be useful to further boost the adaptive immunity in immune-privileged PDAC tumors [64].

Gemcitabine has long been a standard of care for PDAC patients but has limited benefit due to inherent or rapidly acquired resistance. Given the extensive contribution of CXCL12 signaling in drug resistance in PDAC, there is interest in testing if interrupting CXCL12 signaling can resensitize tumors to this or other chemotherapeutics. Our retrospective analyses of the INT-11 trial consisting of 660 pancreatic cancer patients demonstrate that tipifarnib may sensitize PDAC to gemcitabine in a patient population stratified based on the absence of abdominal pain as a surrogate for high CXCL12 expression. Though preliminary, these findings encourage further biomarker-guided investigation of the combination of chemotherapies with CXCL12 inhibition in PDAC. Indeed, other retrospective analyses of the INT-11 trial dataset suggest that the tipifarnib–gemcitabine combination may offer some clinical benefit in patients with metastases limited to CXCL12-rich organs, such as the liver and lymph nodes.

**Figure 3:**
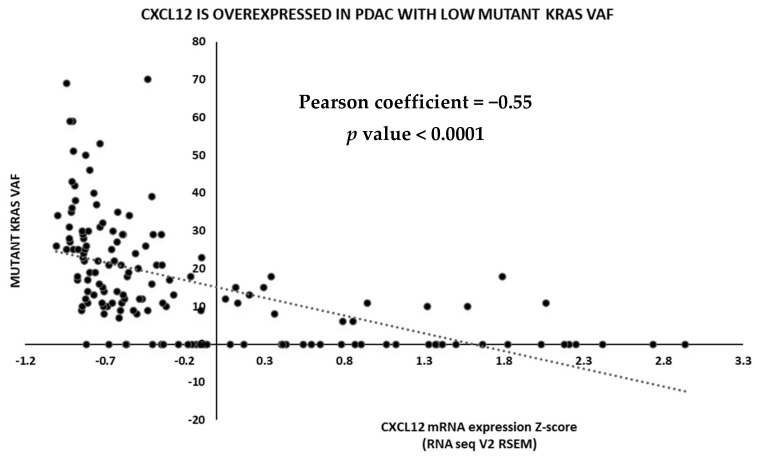
CXCL12 is expressed at higher levels in PDAC bearing low KRAS mutant VAF. CXCL12 expression in TCGA PDAC dataset. PDAC samples were analyzed based on their CXCL12 RNA expression and KRAS VAF. VAF, variant allele frequency; PDAC, pancreatic ductal adenocarcinoma; TCGA, the Cancer Genome Atlas. Pearson coefficient and the associated two-tailed *p* value are depicted on the graph.

**Figure 4:**
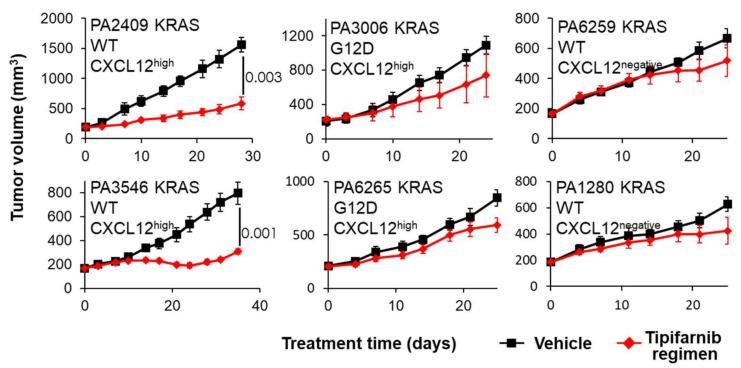
Tipifarnib inhibits tumor growth in WT KRAS CXCL12-producing PDAC PDX models. Mice bearing established subcutaneous PDX tumors were treated with oral tipifarnib BID for 25–35 days. CXCL12^+^/KRAS WT tumors responded well (left panels), whereas CXCL12^+^/KRAS-mutant and CXCL12^−^/KRAS WT tumors were unaffected. PDAC, pancreatic ductal adenocarcinoma; PDX, patient-derived xenograft; WT, wild-type. Unpaired, two-tailed *p* values for PA2409 and PA3546 are depicted on the plots.

A bioinformatic analysis of PDAC datasets from the Cancer Genome Atlas (TCGA) also suggests an intriguing alternative biomarker-based patient selection strategy for CXCL12-directed therapeutics in this disease. KRAS mutation rates are higher in PDAC than any other cancer, so it is often assumed to be an exclusively KRAS-driven disease, but emerging data from large NGS-based studies suggest that 15–25% of cases harbor WT KRAS or display a low variant allele frequency (VAF) of the mutant form [94]. Interestingly, CXCL12 expression was significantly elevated in WT cases and negatively correlated with mutant KRAS VAF in a TCGA PanCancer Atlas cohort (Figure 3). The primary oncogenic signaling pathway downstream of KRAS in PDAC is the MAPK pathway, so taken together with the reported role of CXCL12 in promoting MAPK signaling in PDAC cells, these analyses suggest that CXCL12 may rescue oncogenic MAPK signaling in tumors with WT KRAS or low mutant VAF. Intriguingly, tipifarnib robustly inhibited the growth of CXCL12^+^ PDAC patient-derived xenograft (PDX) tumors, but only in the context of WT KRAS (Figure 4). Although subcutaneous models do not capture the unique tumor microenvironmental and immunosuppressive signatures such as the excessive stromal presence of PDAC, using subcutaneous PDX model in this study is a compromise preliminary solution to the challenge of studying the role of CXCL12 signaling in PDAC. In this study, we used PDX models of a rare subset of PDAC where the malignant cells are a source of the chemokine where we can test the effect of the FTI in the context of a CXCL12-high PDAC tumor mass, but the role of the T-cell immune response cannot be addressed in these nude mice. However, the partial tumor inhibitory response induced by tipifarnib is promising and suggests that CXCL12 silencing with tipifarnib may be of particular benefit in this subset of PDAC patients.

Early clinical data with CXCR4 antagonists are encouraging but their activity needs to be improved upon. AMD3100 (Mozobil) is approved for clinical use but is challenging for repeat dosing [95], so BL-8040 may be more suitable for oncology applications. In addition, PDAC cells frequently co-express CXCR4 and CXCR7; thus, CXCR4 antagonists may only partially disrupt CXCL12 signaling. Indeed, AMD3100 paradoxically activates CXCR7, potentially undermining any advantage of CXCR4 blockade [96]. By contrast, depleting the ligand would turn off signaling through both receptors, resulting in a more robust therapeutic effect. NOX-A12 takes advantage of this strategy by inhibiting CXCL12 but requires repeated intravenous infusion to compensate for the rapid clearance of RNA aptamers. However, tipifarnib is an oral drug that is well-tolerated in PDAC patients, recommending it as a potential best-in-class clinical CXCL12 inhibitor.

Although we have focused on PDAC in this review, mounting evidence suggests that CXCL12 is an important driver of metastasis, immune evasion, and chemoresistance in other solid tumors, including HNSCC [97,98], urothelial cancer [99], and ovarian and breast carcinomas [100,101,102], so it is possible that tipifarnib could find a therapeutic niche across a broad range of CXCL12-associated tumors.

## 7. Conclusions

In summary, CXCL12 produced by the activated fibroblasts of the stroma-dense PDAC plays important roles in acquiring, maintaining, and enhancing several of the classical cancer traits in this difficult to treat malignancy. CXCL12 signaling not only promotes PDAC survival, growth, and spread but also contributes to resistance to chemo- and immunotherapies. Pro-tumorigenic functions of CXCL12 signaling in addition to its role in therapy resistance make this pathway an attractive therapeutic target in PDAC. While early approaches to inhibit CXCL12 pathway were focused on targeting its receptor, CXCR4, new strategies center on targeting CXCL12 itself. Targeting CXCL12 either with a CXCR4 inhibitor (AMD3100) or through CXCL12 antagonist (NOX-A12 aptamer) in combination with cytotoxic or immune modulatory drugs have shown promise in mouse studies and preliminary proof of mechanism studies in clinical trials, but paradoxical activation of a second receptor, CXCR7, by AMD3100 and repeated intravenous infusion of the aptamer required for clinical activity may limit the full potential of these approaches. Considering these limitations, we proposed a novel approach to inhibiting CXCL12 signaling through tipifarnib, a well-tolerated, oral farnesyl transferase inhibitor. We found that tipifarnib inhibits CXCL12 expression in pancreatic stellate cells and reduces tumor growth of KRAS WT PDAC in PDX models, suggesting that tipifarnib may benefit a subset of PDAC patients. Our current studies are focused on the combination of FTIs with chemotherapy and immune-oncology drugs in orthotopic pancreatic cancer models.

## Data Availability

The Pancreatic Cancer dataset used in this study is freely available at cbioportal.org.
